# Distant Supervision for Mental Health Management in Social Media: Suicide Risk Classification System Development Study

**DOI:** 10.2196/26119

**Published:** 2021-08-26

**Authors:** Guanghui Fu, Changwei Song, Jianqiang Li, Yue Ma, Pan Chen, Ruiqian Wang, Bing Xiang Yang, Zhisheng Huang

**Affiliations:** 1 School of Software Engineering Beijing University of Technology Beijing China; 2 Interdisciplinary Laboratory of Digital Sciences Centre national de la recherche scientifique Université Paris-Saclay Orsay France; 3 School of Health Sciences Wuhan University Wuhan China; 4 Department of Artificial Intelligence Vrije Universiteit Amsterdam Amsterdam Netherlands

**Keywords:** deep learning, distant supervision, mental health, crisis prevention

## Abstract

**Background:**

Web-based social media provides common people with a platform to express their emotions conveniently and anonymously. There have been nearly 2 million messages in a particular Chinese social media data source, and several thousands more are generated each day. Therefore, it has become impossible to analyze these messages manually. However, these messages have been identified as an important data source for the prevention of suicide related to depression disorder.

**Objective:**

We proposed in this paper a distant supervision approach to developing a system that can automatically identify textual comments that are indicative of a high suicide risk.

**Methods:**

To avoid expensive manual data annotations, we used a knowledge graph method to produce approximate annotations for distant supervision, which provided a basis for a deep learning architecture that was built and refined by interactions with psychology experts. There were three annotation levels, as follows: free annotations (zero cost), easy annotations (by psychology students), and hard annotations (by psychology experts).

**Results:**

Our system was evaluated accordingly and showed that its performance at each level was promising. By combining our system with several important psychology features from user blogs, we obtained a precision of 80.75%, a recall of 75.41%, and an F1 score of 77.98% for the hardest test data.

**Conclusions:**

In this paper, we proposed a distant supervision approach to develop an automatic system that can classify high and low suicide risk based on social media comments. The model can therefore provide volunteers with early warnings to prevent social media users from committing suicide.

## Introduction

### Background

Mental disorders have become a serious problem worldwide, with over 264 million people experiencing depression disorders [1]. A recent large-scale survey in China showed that the lifetime prevalence of depression and anxiety is 6.9% and 7.6%, respectively [2]. Depression is a leading cause of disability worldwide and contributes greatly to the global burden of diseases [3]. Suicide is the most serious consequence of depression [4]. The latest World Health Organization report showed that close to 800,000 people die by suicide every year. Furthermore, for each suicide, there are more than 20 suicide attempts. Suicides and suicide attempts have a ripple effect that impacts families, friends, colleagues, communities, and societies [5]. Stopping suicides before they are successful is a top priority. With the widespread popularity of the internet and the lack of immediate support in life, people are more inclined to express their emotions—even suicidal thoughts—in web-based communities, such as Weibo (Sina Corporation) and Twitter. Social media users favor these communities due to the anonymity and real-time advantages that they provide [6]. The data from web-based communities are huge in quantity, and it is difficult to analyze these data manually.

Traditional suicide risk assessment studies mainly conduct psychological tests, interviews, and questionnaires, which cost a lot of money. If computer technology can be used to assist suicide risk assessments, we can greatly improve the coverage and efficiency of screening and therefore reduce the number of suicide attempts. In recent years, many deep learning methods have been used for text sentiment analysis. However, these methods require large amounts of labeled data. With regard to the topic of our study, several thousands of comments are generated every day, but only a few (typically, less than 10) are indicative of high suicide risk. It is very time consuming to label so much data, and analyzing low and high suicide risk requires professional knowledge and special training.

In this context, we propose a distant supervision model to reduce the workload of domain experts. We integrated interesting scientific findings from the psychology field into our model. We developed a system that requires no manual annotations and takes into account the feedback of experts to better classify people’s suicide risk levels based on the textual comments published on a Chinese social media platform. To avoid expensive manual data annotations, we used a knowledge graph method to produce approximate annotations, which provided a basis for building a deep learning model. The learning model was further refined by interactions with people with different experiences in psychology (beginners and experts) to generate our three data sets—the free annotation (zero cost), easy annotation (labeled by psychology students), and hard annotation (labeled by psychologists) data sets. We built the following three progressive models to fit these data sets: a bidirectional encoder representations from transformers (BERT)–based model, a fine-tuning model, and the psychology+ model. We obtained a precision of 80.75%, a recall of 75.41%, and an F1 score of 77.98% for the hard annotation data set, which was the hardest data set among the three data sets. This system reports on suicide risk assessments to volunteers from the Tree Hole Rescue Team [7] for web-based crisis prevention in China.

We first introduce the background of our research and discuss related work in the *Introduction* section. In the *Methods* section, we describe the task and introduce our workflow. In the *Results* section, we describe our data sets and a series of experimental results to prove the performance of our method. In the *Discussion* section, we summarize a series of open problems in this study and put forward some thoughts on future research directions. We summarize the whole paper in the final *Conclusion* section.

### Related Work

#### Distant Supervision

Distant supervision is a method for using prior knowledge to generate noise labels (data containing wrong labels), which can help with avoiding a lot of manual labeling. In 2009, Mintz et al [8] first proposed the distant supervision method and used it for relation extraction. They generated a large amount of noise labels by mapping an existing knowledge base to rich unstructured text. Thus, they were able to train a good relationship extractor. Some sentiment classification methods also involve the distant supervision method. Go et al [9] proposed a method for automatically classifying sentiment in Twitter messages. They first used the emoticons in the text to generate labels and then used machine learning algorithms for classification. Purver and Battersby [10] and Suttles and Ide [11] used emoticons and hashtags or emojis in Twitter data to generate noise labels. Sahni et al [12] used half of a data set for comparisons with a baseline model and achieved a 2% to 3% higher accuracy than the baseline model. Camacho-Collados et al [13] proposed a simple, distant, supervision–based postprocessing step for using noisy, user-generated text to learn cross-lingual embeddings. They used English data to train classifiers for other languages. Purver and Battersby [10] used tag symbols and topic tags as weak tag training models. Without human intervention, the models were used to detect multiple types of emotions in Twitter messages. Mohammed et al [14] proposed a novel distant supervision technique for automatically gathering noisy topic category annotations from topically focused streams.

Most related research is based on the same annotation standard and eliminates the differences between different data through distance supervision. In our study, different levels of annotators (from nonprofessional to professional) provided different standards. Our method can be used with different levels of annotators (from basic algorithms to domain experts) to train models and obtain performance improvements to deal with real scenarios.

#### Deep Learning–Based Text Sentiment Analysis

Text sentiment analysis is the task of detecting sentiment information contained in text through a computer program. A basic task in sentiment analysis is classifying the polarity of a given text, that is, whether the expressed opinion in the text is positive, negative, or neutral. In advanced cases, polarity can refer to emotional states such as anger, sadness, and happiness. Sentiment analyses have been applied in marketing, customer service, and clinical medicine. Different from classical text sentiment analysis, our task of classifying high and low suicide risk on the basis of a given text was mainly based on whether users had decided a specific suicide method and a determinate suicide plan. Negative polarity and the emotional states of sadness and anger unnecessarily imply a high risk.

In recent years, many deep learning methods have been used for text sentiment analysis. Kim [15] used the word2vec method to convert sentences into sentence vectors and input the vectors into a convolutional neural network for sentiment analysis and question classification. Kalchbrenr et al [16] designed a dynamic convolutional neural network for binary and multi-class sentiment prediction. The model can handle input sentences of different lengths and can explicitly capture short and long relationships. Dieng et al [17] proposed a recurrent neural network for the sentiment analysis of movie reviews, which could capture the long-term dependency of text sentiment analysis. However, these methods all use word embedding for word vector representation, which cannot solve the problem of polysemy in reality. In 2018, Devlin et al [18] proposed a BERT-based, pretrained language model for dynamically obtaining encoded word vectors according to context. The BERT-based, pretrained language model uses a bidirectional transformer [19] as a feature extractor. Compared to traditional word embedding methods, such as Word2Vec [20-22] and Glove [23], the BERT method is better for capturing the representation of word and sentence levels. The BERT model is one of the most state-of-the-art pretraining models. Our final model was modified based on BERT, integrated the psychology features (Textbox 1) of users, and provided a more comprehensive suicide risk detection system.

**Psychology features and their definitions.**

**Self-description length**

- The number of bytes that a user’s homepage simply introduces to them

**Weibo originality rate**

- The ratio of the amount of original Weibo data to the total amount of Weibo data

**Weibo link rate**

- The ratio of the number of linked Weibo pages to the total number of Weibo pages

**Weibo interaction rate**

- The average number of mentions of other people in a user’s Weibo account

**Collective attention rate**

- The average number of times each Weibo user uses first-person plural terms

**Self-focus rate**

- The average number of times each Weibo user uses first-person singular terms

**Nighttime activity rate**

- The ratio of the number of active users on Weibo from 10 PM to 6 AM to the total number of Weibo users

**Social activity rate**

- The ratio of the number of mutual attentions and the number of followers that a Weibo user has to those of other users

**Willingness to express rate**

- The ratio of the number of a user’s Weibo comments to the number of their likes

**Social support rate**

- The ratio of the number of a user’s Weibo comments to the total number of Weibo comments

## Methods

### Study Design

**Figure 1:**
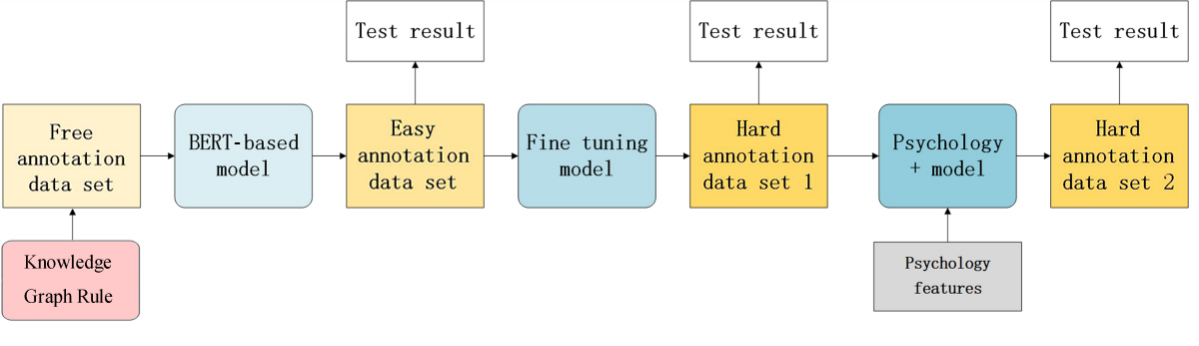
Flowchart of our method. As the labeling process became stricter, we continued to improve our model's performance. BERT: bidirectional encoder representations from transformers.

As previously mentioned, the information for classifying high and low suicide risk in our task is different from those used in the classical sentiment analysis tasks. In this study, to avoid the high cost of creating manually annotated data, we used a distant supervision approach that does not require manual annotations. Domain experts can use this approach to provide a small amount of annotations, which provides a basis for further improving a model by taking into account the experts’ feedback. The flowchart of our method is shown in Figure 1.

The method was divided into two parts—automated annotation generation (via the knowledge graph rules in Figure 1) and deep learning–based classification (the three models in Figure 1).

For automated annotation generation, a set of semantic rules was constructed based on an ontology for the field of crisis prevention to generate the free annotation data set. These automated (possibly erroneous) annotations were then used to supervise the deep learning models.

We then build a BERT-based model based on the free annotation data set. This model was used to classify new texts that were further corrected by psychology students to generate the easy annotation data set. This data set had comparable amounts of high and low crisis risk data. It should be noted that without the assistance of a computer algorithm, it would have been a massive challenge for humans to provide such a balanced data set because the percentage of high-risk messages was quite low, as mentioned above. We used the easy annotation data set to fine-tune this basic learning model and develop the fine-tuning model, which took into account the knowledge in the easy annotations. In parallel, a psychology expert assisted with providing the hard annotation data set, which is of much smaller size than the easy annotation data set due to its cost. Finally, we improved our model by using the hard annotation data set and extra psychology features to obtain the final psychology+ model.

Our models continually fitted 3 data sets. These three data sets were labeled by psychology practitioners of different levels. As the standards for the labeling process gradually became stricter, the models became more accurate. The final model (psychology+ model) combined the prior knowledge obtained from the precedent models and fused prior domain knowledge. Improved performance was achieved under the premise of using a small amount of manually annotated data.

### Task Definition

Given a textual sentence (*s*) published on a web-based media platform by a user (*u*), our task was to predict if the user (*u*) has a high suicide risk. If this is the case, a crisis intervention will be provided to save the user’s life. This is a challenging task, and even a person with professional knowledge has to be careful to avoid mistakes. Furthermore, the huge amount of comments produced daily makes manual analysis impossible and costly.

With regard to the definitions of high and low risk, Huang et al [24] proposed a grading standard based on the certainty of planned suicide methods and the urgency of the action, thereby obtaining 10 risk levels, as shown in Textbox 2. We defined level 6 and above as “high risk” and the rest as “low risk.” Furthermore, social media may contain a lot of irrelevant comments. Therefore, we created level 0 and defined it as “no risk.”

**Suicide risk levels and judgement criteria.**

**Level 10**

- Suicide may be ongoing

**Level 9**

- Suicide method has been determined
- Person may commit suicide in the near future

**Level 8**

- Suicide has been planned
- Date of suicide has been roughly determined

**Level 7**

- Suicide method has been determined but not the suicide date

**Level 6**

- Suicide method is planned
- Suicide method is unknown

**Level 5**

- Strong suicide desire
- Unclear mode of suicide

**Level 4**

- Suicide wish has been expressed
- Suicide method and plan are unclear

**Level 3**

- Strong survival pain
- No expression of suicidal wish

**Level 2**

- The pain of survival has been clearly expressed
- No suicidal desire

**Level 1**

- Expression of survival pain
- Expression of suicide desire

**Level 0**

- No expression of survival pain

### Knowledge Graph and Reasoning Rules

**Figure 2:**
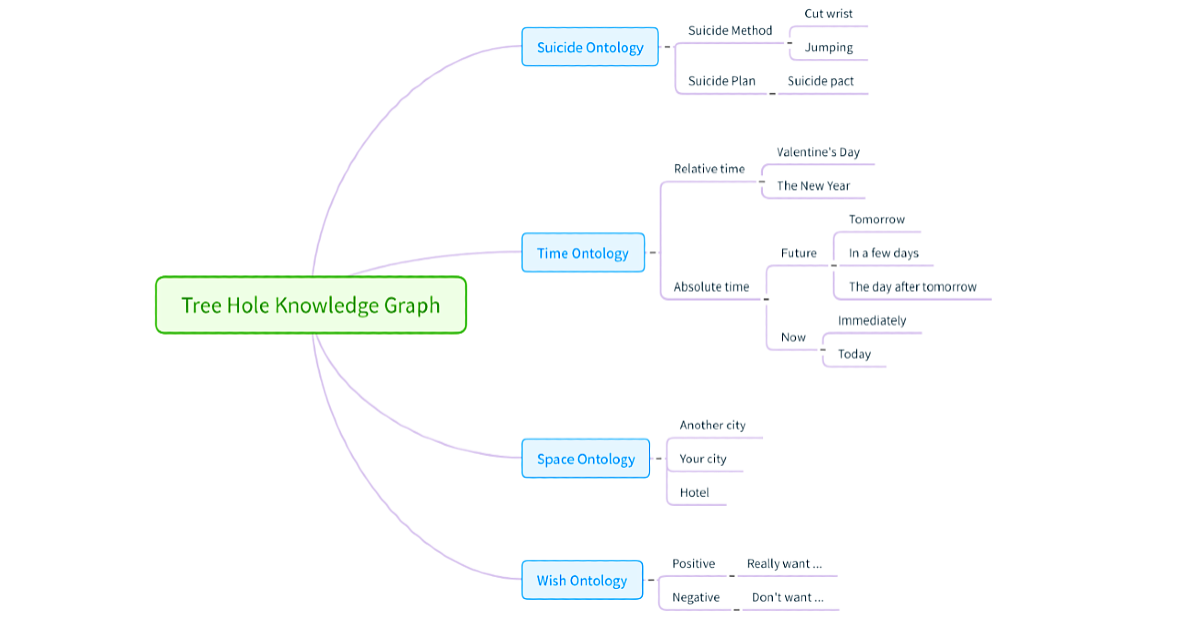
An excerpt of the Tree Hole knowledge graph.

We created a knowledge graph that was used to construct a free annotation data set. The Tree Hole knowledge graph contains four independent ontologies—the suicide ontology, time ontology, space ontology, and wish ontology. The suicide ontology consists of two major categories—suicide methods (eg, cutting the wrist and burning charcoal) and suicide plans (eg, buying drugs and meeting with suicide partners). The time ontology covers absolute time concepts, such as calendar days and holidays, and relative time concepts, such as the present, future, and past. The space ontology describes related concepts of spatial geography. The wish ontology was mainly used to analyze the subjective suicidal wishes of a specific group of people and to exclude them from people without subjective suicidal wishes. An excerpt of the Tree Hole knowledge graph can be found in Figure 2.

Once the knowledge graph was created, we constructed prolog rules based on the definite clause grammar (DCG) and DCG transformation rules. To take into account the domain knowledge for reasoning and determining the risk level, the DCG rules integrate the relevant conceptual information from the Tree Hole knowledge graph. For example, the definition for suicide risk level 8 is that the suicide plan has been determined and the suicide date has been roughly determined. This can be formally described by the logic program rules in Textbox 3, which use two predicates—*rdfsSubClassOf* and *uninterestedText*. The predicate *rdfsSubClassOf* was used to decide if a textual fragment mentions some sort of suicide method (or time) concept of *suicideOntology* (or *timeOntology*) in the Tree Hole knowledge graph, and *uninterestedText* was used to verify that such a mention was not negated by a negative expression in the text. More details can be found in other studies [24,25].

**Logic program rules for suicide risk level 8.**

**Program rules**

- statement(suicideRisk(8,[Plan, Time]))→ uninterestedText(_L1), rdfsSubclassOf(Time,future,timeOntology), uninterestedText(_L2), rdfsSubclassOf(Plan, suicidePlan, suicideOntology), uninterestedText(_L3)
- statement(suicideRisk(8, [Plan, Time]))→ uninterestedText(_L1), rdfsSubclassOf(Plan, suicidePlan, suicideOntology), uninterestedText(_L2), rdfsSubclassOf(Time, future, timeOntology), uninterestedText(_L3).

### BERT-Based Model

**Figure 3:**
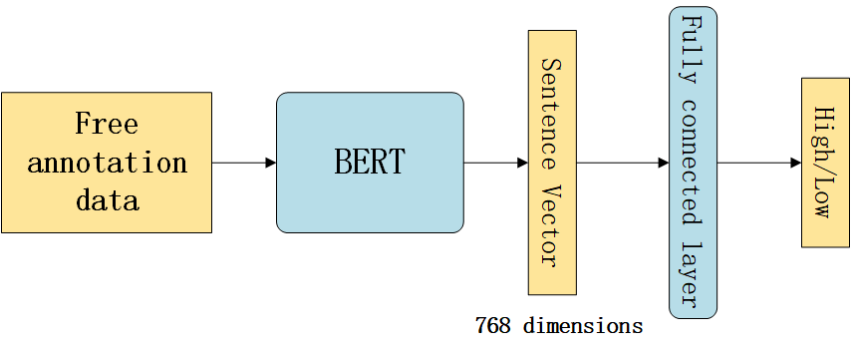
Architecture of our BERT-based model. BERT: bidirectional encoder representations from transformers .

We used the data set generated by Tree Hole knowledge graph method to build the BERT-based model. We used BERT to obtain sentence vectors from free annotation data. The size of each sentence vector was 768 dimensions. We added a dropout function to this sentence vector to avoid overfitting. Afterward, we added a fully connected layer to classify comments that were indicative of high and low suicide probabilities. We used the sigmoid function as the activation function of the output layer. The parameters of the BERT layer and the fully connected layer participated in the training at the same time. The architecture of model 1 is shown in Figure 3.

### Fine-Tuning Model

**Figure 4:**
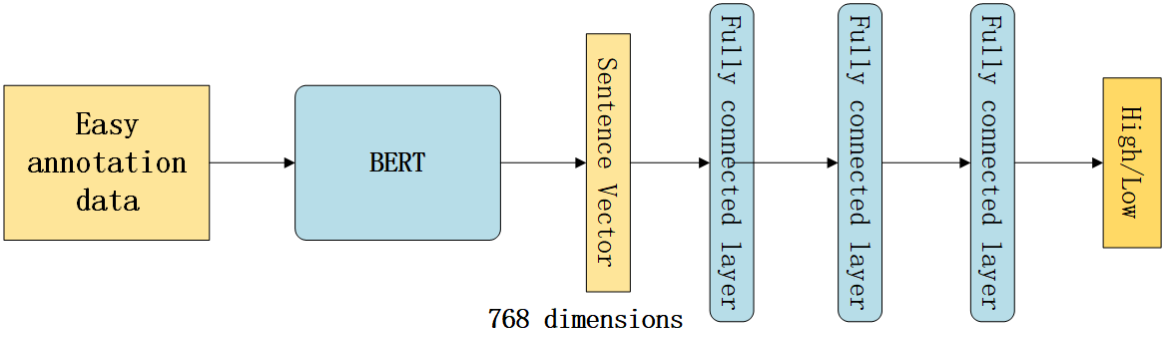
Architecture of our fine-tuning model. BERT: bidirectional encoder representations from transformers.

We used BERT-trained (the first model) to obtain 768D sentence vectors from the easy annotation data set. We added 3 fully connected layers to the sentence vectors. The input and output of the first 2 fully connected layers had 768 dimensions, and we used the ReLU function [26] as their activation function. We also added a dropout function and batch normalization function [27] after each activation function to prevent overfitting. We used the sigmoid function as the activation function of the output layer. Different from the first model, the fine-tuning model did not train the BERT layer but only trained the previous fully connected layer. The structure of model 2 is shown in Figure 4.

### Psychology+ Model

This model combined deep learning and psychology features. According to a psychology study, people with a high suicide risk have a higher degree of self-concern than those with a low suicide risk, that is, they may be more focused on themselves rather than their surroundings [28]. Social network behaviors can reflect individuals’ personality traits [29]. Gosling et al [30] found that people with extraverted personality traits use social networks more frequently, have higher engagement, and are more active compared to introverts. To take advantage of these scientific findings, we built the psychology+ model, as described below.

**Figure 5:**
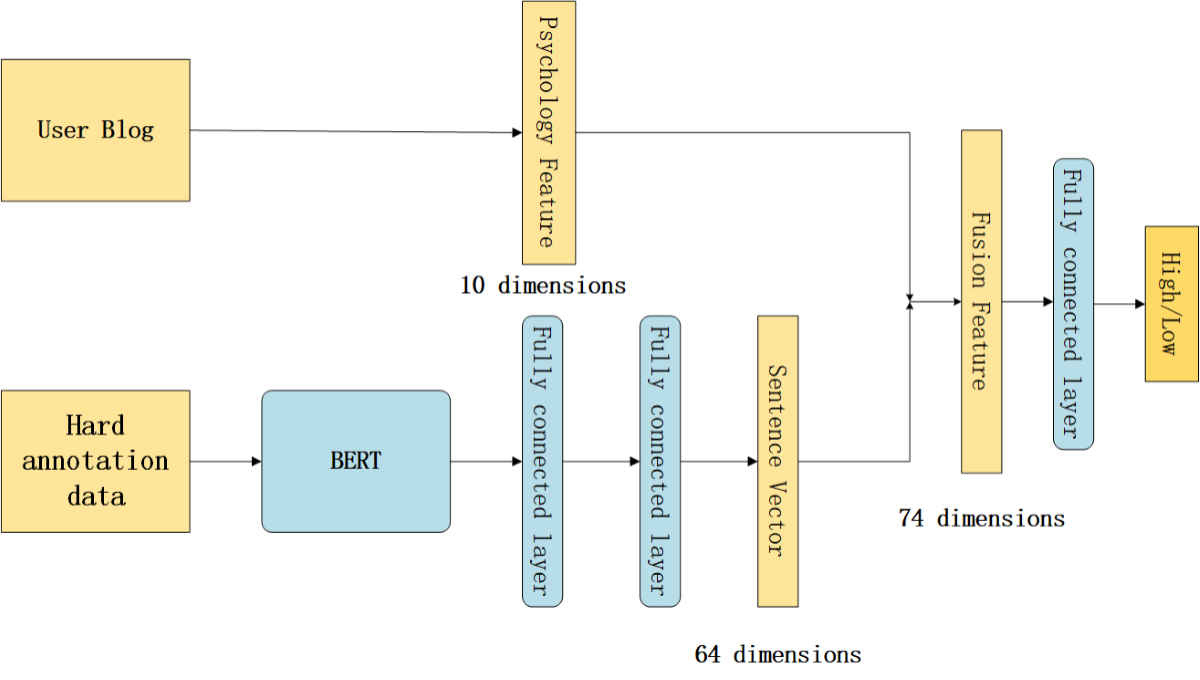
Architecture of our psychology+ model. BERT: bidirectional encoder representations from transformers.

We analyzed the 10 psychology features defined in Textbox 1. According to psychological research, people with a high and low suicide risk have significantly different behaviors with regard to these 10D features [28-32]. We further analyzed and obtained Weibo users’ psychology features and integrated them into our final model. These 10 features were represented as a 10D vector. Afterward, we used the BERT-trained model (the first model) to obtain 768D sentence vectors from hard annotation data. We used 2 fully connected layers to reduce the sentence vector to 64 dimensions. Our choice of using a 64D vector was based on experiments. We experimented with 256, 128, 64, 32 dimensions, and the best results were obtained with 64 dimensions. We combined the 64D sentence vector and 10D psychological feature vector to form a 74D vector. Finally, we used the fully connected layer as a classifier and the sigmoid function as the activation function to classify high and low suicide risk. This model structure is shown in Figure 5.

## Results

### Data Set

In this study, data were obtained from the comments of the Zoufan Weibo account [33]. In addition, we performed the following preprocessing methods for the data sets: the removal of duplicate data, the removal of meaningless emojis, and the removal of too short sentences (≤5 words). As previously explained, we constructed the following four data sets (our annotation data sets can be made available upon request for research purposes):

- the free annotation data set, which was generated by using knowledge graph method
- the easy annotation data set, which was labeled by a psychology student
- hard annotation data sets 1 and 2, which were labeled by a psychologist

These data sets were used to train and test our models. The data distribution can be seen in Table 1.

For the psychology+ model, the psychology features were extracted from each user’s Weibo page [34].

**Table 1:** Comment distributions in each data set.

Data set	Comments indicating a high suicide risk, n	Comments indicating a low suicide risk, n
Free annotation data set	3630	3220
Easy annotation data set	6659	8657
Hard annotation data set 1	813	645
Hard annotation data set 2	599	601

### Evaluation

We evaluated the three learning models that were built based on the automatically generated annotation data set. We found that the free and easy annotation data sets resulted in a simple classification task that could be solved well by the BERT-based model and the fine-tuning model, respectively. However, the hard annotation data set resulted in a much harder task for which our psychology+ model could achieve a promising performance.

### Experimental Setup

For the basic BERT-based model, we used the pretrained Chinese model released by Google. The model uses a 12-layer transformer with about 110 million parameters. The optimizer uses the Adam method [35], and the learning rate was set to 0.00002. The maximum length of inputted characters is 128. The batch sizes of the training set, validation set, and test set were 8, 16, and 16, respectively. At the fully connected layer, the dropout was set to 0.1. We built our model by using a GTX 2080Ti graphics processing unit, and the deep learning framework we used was PyTorch [36]. In the evaluation experiments, we used F1 scores, precision, recall, and the accuracy of high-risk classifications as the evaluation metrics. The F1 score is the harmonic mean of precision and recall, and it was the most important evaluation metric in our study.

### Evaluation of the BERT-Based Model

We performed fivefold cross-validation for 6850 comments (3630 comments indicating a high suicide risk and 3220 indicating a low suicide risk; Table 1) and thus obtained 5480 comments for training and 1370 comments for testing. We report in Table 2 the evaluation results of the model that was based on the free annotation data set and the easy annotation data set.

We found that the values of all evaluations metrics for the free annotation data set were higher than 0.98. This shows that this simple model can simulate the knowledge graph–based approach well. The model’s performance based on the easy annotation data set was lower, particularly in terms of precision (0.9899 vs 0.8367), indicating that the model needs further improvement to analyze behavior just as well as psychology students for the annotation task.

**Table 2:** Bidirectional encoder representations from transformers–based model.

Data set	F1 score	Precision	Recall	Accuracy
Free annotation data set	0.9864	0.9899	0.9829	0.9862
Easy annotation data set	0.9111	0.8367	0.9998	0.9151

### Evaluation of the Fine-Tuning Model

According to the fivefold cross-validation, we separated 15,316 comments (6659 comments indicating a high suicide risk and 8657 indicating a low suicide risk; Table 1) into the training set or the test set, which contained 12,252 and 3064 comments, respectively. We also evaluated the performance of the model based on hard annotation data sets 1 and 2.

The results can be seen in Table 3. Clearly, by fine-tuning the model, the performance of the model improved (compared to the BERT-based model) in terms of F1 score (0.9111 vs 0.9214), precision (0.8367 vs 0.9241), and accuracy (0.9151 vs 0.9218). However, the recall value dropped a little bit (0.9998 vs 0.9282).

In contrast to the performance of the model based on the easy annotation data set, the results for the 2 hard annotation data sets were unsatisfactory (<0.6 in most cases). This meant that the model needed further improvement. Intuitively, we could have performed a similar fine-tuning process with the hard annotations. However, as seen in Table 1, the hard annotation data sets were much smaller than the easy annotation data set. This was due to the high cost of the hard annotations. Therefore, we created the psychology+ model by taking into account certain psychological knowledge in order to avoid the requirement of large amounts of annotated data.

**Table 3:** Fine-tuning model.

Data set	F1 score	Precision	Recall	Accuracy
Easy annotation data set	0.9214	0.9241	0.9282	0.9218
Hard annotation data set 1	0.7281	0.5815	0.9734	0.5942
Hard annotation data set 2	0.6753	0.5131	0.9877	0.5239

### Evaluation of the Psychology+ Model

According to fivefold cross-validation, we separated 1458 comments (813 comments indicating a high suicide risk and 645 indicating a low suicide risk from hard annotation data set 1; Table 1) into the training set or the validation set, which contained 1167 and 291 comments, respectively. Hard annotation data set 2 was reserved mainly for testing. We report the evaluation results for these two hard annotation data sets in Table 4. We obtained an accuracy of 78.68% for hard annotation data set 2. Compared to the fine-tuning model, the F1 score of the psychology+ model based on hard annotation data sets 1 and 2 improved by 7.88% and 10.45%, respectively.

**Table 4:** Psychology+ model.

Data set	F1 score	Precision	Recall	Accuracy
Hard annotation data set 1	0.8069	0.8067	0.8072	0.8105
Hard annotation data set 2	0.7798	0.8075	0.7541	0.7868

## Discussion

### Principal Findings

#### Summary of Findings

In this study, we examined three types of annotation data sets—the free annotation, easy annotation, and hard annotation data sets. As seen in Table 2 and Table 3, free annotations and easy annotations can be generated by deep learning models of high quality. Even though we achieved promising results from the hard annotation data sets, the quality of the results is still imperfect. Indeed, our task was exposed to some challenges due to several characteristics of the data.

We consider the F1 score to be the most important evaluation metric. In follow-up work, we will invite more volunteers to manually evaluate our model based on actual situations.

#### Obscure Expression

**Figure 6:**
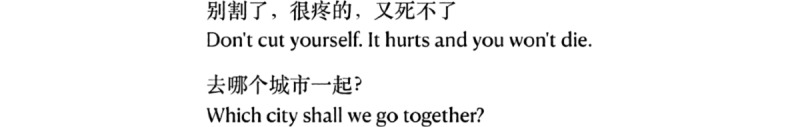
Examples of comments that express a high suicide risk in an obscure way.

With regard to the two sentences in Figure 6, without considering the context of the task, neither of these two statements are indicative of a high risk of suicide. However, due to the nature of our data source, both sentences were classified as high risk by psychological experts. The first sentence expresses the result of someone cutting their wrist, but in terms of our context, the sentence was used to comment on a suicide attempt (cutting wrist), indicating that the attempt has been taken. Therefore, a prevention intervention should be provided. The second sentence is about making an appointment with other people to die together. In the context of our data source, it can be considered that the date of the suicide was about to be determined, resulting in a high risk level of between 7 and 8.

#### Ultrashort Text

**Figure 7:**
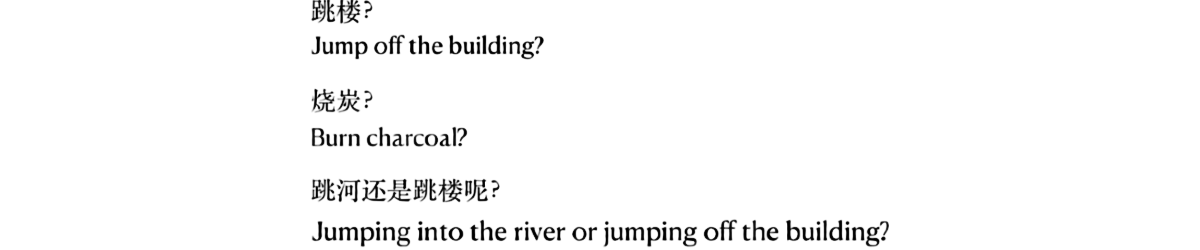
Ultrashort texts that are indicative of different suicide risk levels.

Short texts can also indicate a high suicide risk. For example, for the three short sentences in Figure 7, the first two are indicative of a low suicide risk, but the third is indicative of a high suicide risk. The is because the first two expressions indicate that the plan is still under consideration. However, the third indicates that the plan has been restricted to two possible suicide methods. Such ultrashort texts are difficult to classify, and successful classification needs the combination of more specific context and domain knowledge.

#### Long Text With Contradictions

**Figure 8:**
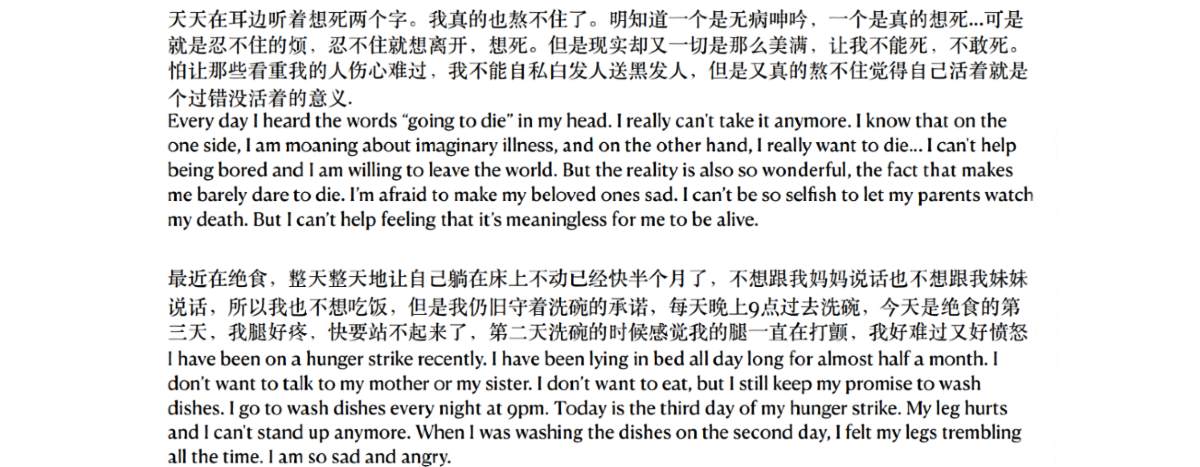
Examples of long texts for which the suicide intention level is hard to capture.

Some people express their feelings by telling their own stories, as illustrated by the examples given in Figure 8. These long, contradictory, redundant, and possibly incomprehensible expressions and sentences also make classification difficult.

### Conclusion

In this paper, we proposed a distant supervision approach to develop an automatic system that can classify high and low suicide risk based on social media comments. We constructed 3 data sets of different levels (free, easy, and hard) via interactions with psychologists who were assisted by our models.

Although a deep learning model has excellent performance in different domains, it requires a lot of annotations to train a reliable model. Per our study, ordinary people cannot accurately label data. Only people who have been professional trained can accurately classify the suicide risk expressed in comments in accordance with standard methods. This makes it difficult to obtain large-scale annotations. Per our processing steps, we first used a basic algorithm to generate annotations for training the baseline model. Afterward, we invited people with different psychology knowledge levels to provide a small number of annotations. Then, based on domain knowledge, we extracted users' multidimensional psychological features and integrated them into our final model (the psychology+ model). Our operating steps greatly improved the efficiency of our model. Only 1458 professional labels were required to train a model that could analyze real situations. It would have been impossible to train a reliable model with just over 1000 data points if we did not conduct the previous steps.

In future work, we will combine actual work experiences and cooperate with psychologists to propose more suitable suicide classification standards and provide immediate warnings for upcoming emergencies. Although our model could meet actual standard requirements, its run time was relatively slow (274 comments/minute) due to the large number of model parameters. Further, although the model’s efficiency could meet people’s daily needs, we still hope to develop a more lightweight model for dealing with certain data produced in special situations, such as short-term, large-scale comments.

## Abbreviations

BERT: bidirectional encoder representations from transformers
DCG: definite clause grammar
