# Correction: Distant Supervision for Mental Health Management in Social Media: Suicide Risk Classification System Development Study

**DOI:** 10.2196/33229

**Published:** 2021-09-03

**Authors:** Guanghui Fu, Changwei Song, Jianqiang Li, Yue Ma, Pan Chen, Ruiqian Wang, Bing Xiang Yang, Zhisheng Huang

**Affiliations:** 1 School of Software Engineering Beijing University of Technology Beijing China; 2 Interdisciplinary Laboratory of Digital Sciences Centre national de la recherche scientifique Université Paris-Saclay Orsay France; 3 School of Health Sciences Wuhan University Wuhan China; 4 Department of Artificial Intelligence Vrije Universiteit Amsterdam Amsterdam Netherlands

In “Distant Supervision for Mental Health Management in Social Media: Suicide Risk Classification System Development Study” (J Med Internet Res 2021;23(8):e26119), one error was noted.

In the originally published article, the email address of the corresponding author was incorrect.

The email address was originally published incorrectly as *“00009312@whu.wdu.cn”*.

This has been corrected to *“00009312@whu.edu.cn”*.

The correction will appear in the online version of the paper on the JMIR Publications website on September 3, 2021, together with the publication of this correction notice. Because this was made after submission to PubMed, PubMed Central, and other full-text repositories, the corrected article has also been resubmitted to those repositories.

